# Difference of Cerebrospinal Fluid Biomarkers and Neuropsychiatric Symptoms Profiles among Normal Cognition, Mild Cognitive Impairment, and Dementia Patient

**DOI:** 10.3390/ijms25073919

**Published:** 2024-03-31

**Authors:** Ching-Chi Hsu, Shiow-Ing Wang, Hong-Chun Lin, Eric S. Lin, Fan-Pei Yang, Ching-Mao Chang, James Cheng-Chung Wei

**Affiliations:** 1 Board of Directors, Wizcare Medical Corporation Aggregate, Taichung 404, Taiwan; chi7hsu@gmail.com; 2 International Intercollegiate Ph.D. Program, National Tsing Hua University, Hsinchu 300, Taiwan; 3 Center for Health Data Science, Department of Medical Research, Chung Shan Medical University Hospital, Taichung 402, Taiwan; shiowing0107@gmail.com; 4 Institute of Medicine, Chung Shan Medical University, Taichung 402, Taiwan; 5 Center for Traditional Medicine, Taipei Veterans General Hospital, Taipei 112, Taiwan; linhcdr@gmail.com; 6 Institute of Traditional Medicine, National Yang Ming Chiao Tung University, Taipei 112, Taiwan; 7 Department of Economics, National Tsing Hua University, Hsinchu 300, Taiwan; slin@mx.nthu.edu.tw; 8 EMBA/MBA/MFB/MPM/HBA Programs, National Tsing Hua University, Hsinchu 300, Taiwan; 9 Department of Foreign Languages and Literatures, National Tsinghua University, Hsinchu 300, Taiwan; fbyang@gmail.com; 10 Department of Oral and Maxillofacial Radiology, Graduate School of Dentistry, Osaka University, Osaka 565-0871, Japan; 11 School of Medicine, College of Medicine, National Yang Ming Chiao Tung University, Taipei 112, Taiwan; 12 Department of Nursing, Chung Shan Medical University, Taichung 402, Taiwan; 13 Department of Allergy, Immunology and Rheumatology, Chung Shan Medical University Hospital, Taichung 402, Taiwan; 14 Graduate Institute of Integrated Medicine, China Medical University, Taichung 402, Taiwan

**Keywords:** mild cognitive impairment, dementia, Alzheimer’s disease, Amyloid-β, P-tau181, T-tau, cerebrospinal fluid biomarker, neuropsychiatric inventory questionnaire

## Abstract

The delineation of biomarkers and neuropsychiatric symptoms across normal cognition, mild cognitive impairment (MCI), and dementia stages holds significant promise for early diagnosis and intervention strategies. This research investigates the association of neuropsychiatric symptoms, evaluated via the Neuropsychiatric Inventory (NPI), with cerebrospinal fluid (CSF) biomarkers (Amyloid-β42, P-tau, T-tau) across a spectrum of cognitive states to enhance diagnostic accuracy and treatment approaches. Drawing from the National Alzheimer’s Coordinating Center’s Uniform Data Set Version 3, comprising 977 individuals with normal cognition, 270 with MCI, and 649 with dementia. To assess neuropsychiatric symptoms, we employed the NPI to understand the behavioral and psychological symptoms associated with each cognitive category. For the analysis of CSF biomarkers, we measured levels of Amyloid-β42, P-tau, and T-tau using the enzyme-linked immunosorbent assay (ELISA) and Luminex multiplex xMAP assay protocols. These biomarkers are critical in understanding the pathophysiological underpinnings of Alzheimer’s disease and its progression, with specific patterns indicative of disease stage and severity. This study cohort consists of 1896 participants, which is composed of 977 individuals with normal cognition, 270 with MCI, and 649 with dementia. Dementia is characterized by significantly higher NPI scores, which are largely reflective of mood-related symptoms (*p* < 0.001). In terms of biomarkers, normal cognition shows median Amyloid-β at 656.0 pg/mL, MCI at 300.6 pg/mL, and dementia at 298.8 pg/mL (*p* < 0.001). Median P-tau levels are 36.00 pg/mL in normal cognition, 49.12 pg/mL in MCI, and 58.29 pg/mL in dementia (*p* < 0.001). Median T-tau levels are 241.0 pg/mL in normal cognition, 140.6 pg/mL in MCI, and 298.3 pg/mL in dementia (*p* < 0.001). Furthermore, the T-tau/Aβ-42 ratio increases progressively from 0.058 in the normal cognition group to 0.144 in the MCI group, and to 0.209 in the dementia group (*p* < 0.001). Similarly, the P-tau/Aβ-42 ratio also escalates from 0.305 in individuals with normal cognition to 0.560 in MCI, and to 0.941 in dementia (*p* < 0.001). The notable disparities in NPI and CSF biomarkers among normal, MCI and Alzheimer’s patients underscore their diagnostic potential. Their combined assessment could greatly improve early detection and precise diagnosis of MCI and dementia, facilitating more effective and timely treatment strategies.

## 1. Background

Mild cognitive impairment (MCI) represents an intermediate stage situated between the expected cognitive decline associated with normal aging and the more pronounced decline observed in dementia [1]. It encompasses issues with memory, language, thinking, and judgment that surpass typical age-related changes [2]. Alzheimer’s disease (AD), the predominant cause of dementia, is a progressive neurological disorder that impairs memory, thinking abilities, and the capacity to perform simple tasks [3,4]. Recent research indicates that the global prevalence of MCI among individuals aged 50 and older is 15.56% [5], with regional prevalences of 19.7% in Europe, 20.7% in the US, 18.2% in the East Asia Pacific [6], and 15.5% in China [7]. The prevalence of AD ranges from 3–5.05% in Europe [8,9], 5.6–6.5% in the US [10], to 3.8–4.0% in China [11,12]. The diagnosis of MCI is contingent upon the observable decline in cognitive capabilities, including memory and thinking skills, as reported by the individual or noted by others [13].

The progression from MCI to AD is thought to be precipitated by the accumulation of the cerebrospinal fluid (CSF) biomarkers such as Amyloid-β plaques, P-tau181, and T-tau tangles within the brain, which are key indicators of AD’s pathology [4,14]. Amyloid-β peptides aggregate into neurotoxic plaques surrounding neurons, leading to inflammation and neuronal dysfunction [15,16,17,18]. P-tau181 indicates an abnormal phosphorylation of tau proteins, resulting in neurofibrillary tangles that disrupt neuronal function. Concurrently, elevated levels of T-tau in cerebrospinal fluid reflect the degree of neuronal damage, serving as markers of advanced neurodegeneration [19,20,21]. The presence and levels of these proteins play a crucial role in monitoring the progression of the disease, particularly among individuals with MCI who are at increased risk of developing AD [15,17,18]. Moreover, factors such as the presence of ApoE4, brain inflammation, oxidative stress, and metabolic imbalances may also contribute to the worsening of this condition [22,23,24,25].

The early identification of AD is pivotal for implementing interventions promptly, potentially decelerating the disease’s progression and alleviating its symptoms [26]. The Neuropsychiatric Inventory Questionnaire (NPI) frequently identifies neuropsychiatric symptoms in AD and MCI, which correlate with an accelerated cognitive decline [27]. Moreover, CSF biomarkers, including Amyloid-β, P-tau181, and T-tau, play a crucial role in AD’s pathogenesis [28]. For individuals diagnosed with either MCI or AD, the predominant focus of current treatment approaches is on alleviating symptoms through a combination of pharmacological treatments, cognitive rehabilitation practices, and structured physical exercise programs [29,30]. It is essential, however, to recognize that a definitive cure for AD has not yet been discovered [31]. Further complicating this issue is the increasing global incidence and prevalence of both MCI and AD, highlighting the critical need for ongoing research and the development of novel intervention strategies [32,33,34,35].

Previous research has largely focused on isolated assessments of the NPI or CSF biomarkers [14,36,37]. Yet, the limited investigation into the correlation between NPI scores and CSF biomarker concentrations calls for additional longitudinal studies [38], which could reveal novel diagnostic markers or treatment options for individuals transitioning from MCI to AD [39]. Therefore, our study seeks to fill this research void by analyzing variations in NPI and essential CSF biomarkers among individuals with normal cognition, MCI, and AD. Concentrating on subjects across these cognitive states, our research contrasts NPI scores with key CSF biomarkers—Amyloid-β, P-tau181, and T-tau—obtained from the National Alzheimer’s Coordinating Center (NACC). The principal goal is to delineate the distinctions highlighted by the NPI and these select CSF biomarkers. Through exploring the relationship between neuropsychiatric symptoms, as measured by the NPI, and the levels of significant CSF biomarkers among individuals with normal cognition, MCI, and dementia, our study aims to probe this previously uncharted territory. We anticipate the identification of significant differences in NPI and CSF biomarker levels across these groups, which could provide crucial insights for enhancing early diagnosis and treatment strategies. Our investigation relies on a thorough analysis of both NPI and CSF biomarker levels, aiming to generate findings that have meaningful implications for both future research and clinical practice.

## 2. Results

### 2.1. Study Population Characteristics

A total of 1896 participants were eligible for the present study. The basic characteristics of the participants stratified by cognition status are shown in Table 1. According to the study design, a total of 977 cases of normal cognition, 270 cases of MCI, and 649 cases of dementia were acquired.

Compared to normal cognition, dementia subjects were more likely to be male, lower education level, married, living with others, and dependent (all *p* < 0.001). However, they exhibit lower rates of comorbidities such as arthritis, sleep disorders, or cancers (all *p* < 0.001). Furthermore, there are notable variations among the three groups regarding their current usage of NSAIDs, antipsychotic agents, hormone therapy, antihypertensive medications, lipid-lowering medication, diabetes medication and antidepressants (all *p* < 0.05).

### 2.2. Performance of Neuropsychological Symptoms

**Figure 1:**
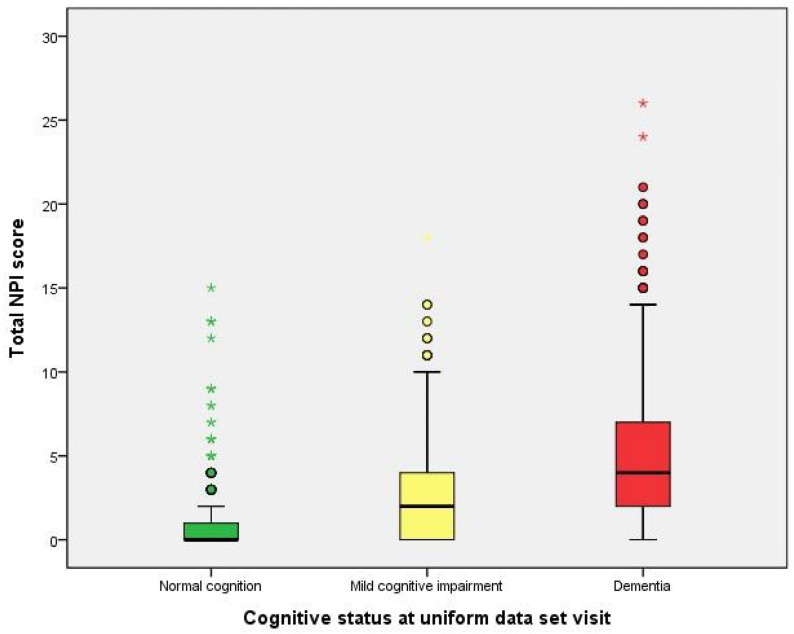
Distribution of total NPI score stratified by cognitive status. o (circle): Mild outliers, data points that are more extreme than than Q3 + 1.5 * IQR,, but are not extreme outliers. * (asterisk): Extreme outliers, data points that are more extreme than Q3 + 3 * IQR.

**Figure 2:**
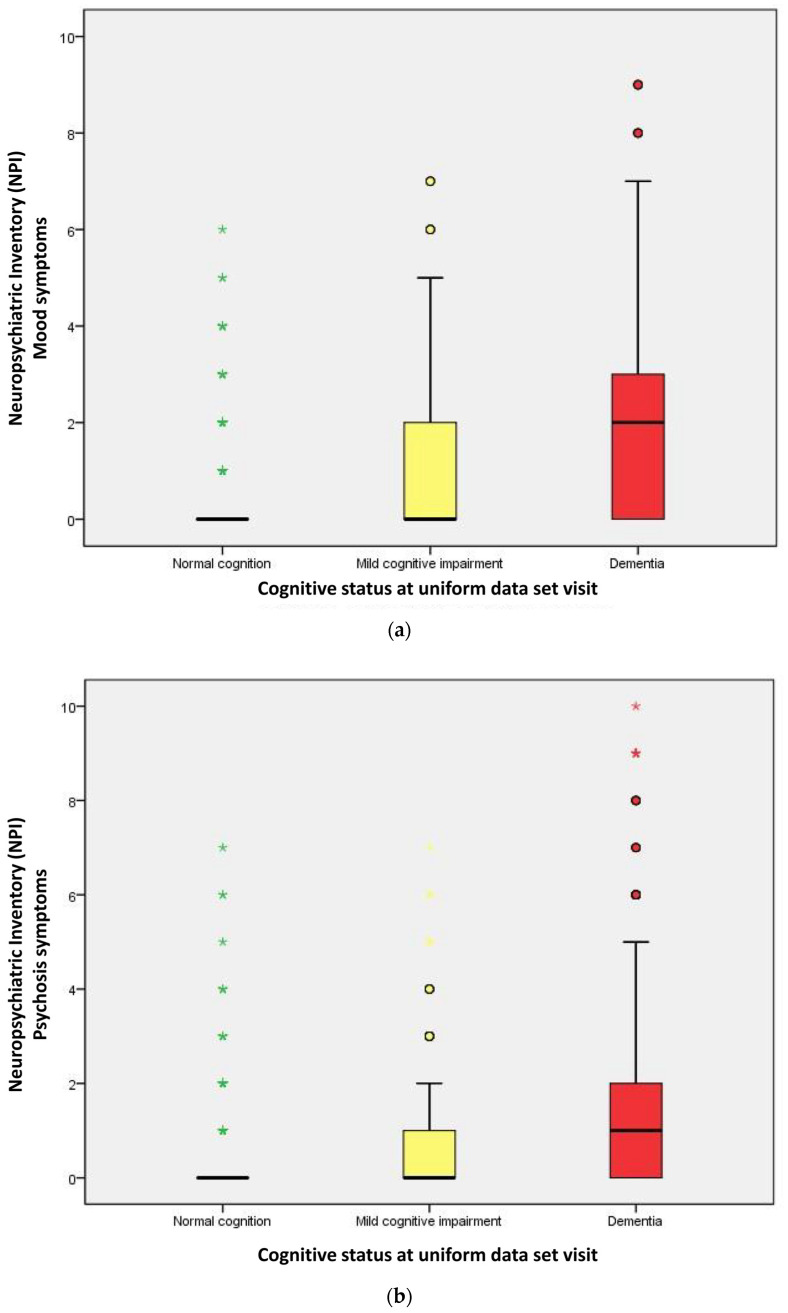

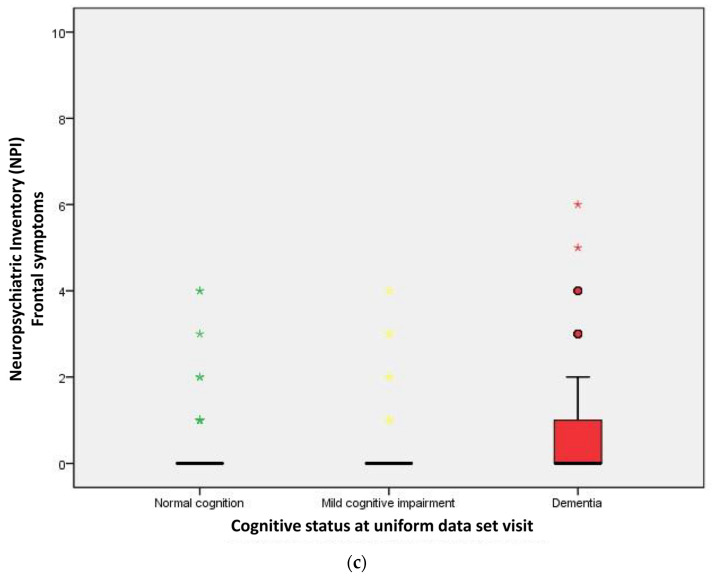
Distribution of NPI-group score stratified by cognitive status. (**a**) Mood symptoms, (**b**) Psychosis symptoms, (**c**) Frontal symptoms. o (circle): Mild outliers, data points that are more extreme than than Q3 + 1.5 * IQR, but are not extreme outliers. * (asterisk): Extreme outliers, data points that are more extreme than Q3 + 3 * IQR.

Table 2 describes the performance of neuropsychological symptoms (NPI-Q) and CSF biomarkers stratified by cognitive level. The distribution of neuropsychiatric behavioral symptoms and three groups of NPI between cognitive levels are significantly different (all *p* < 0.001). These symptoms were significantly more prevalent in dementia subjects as compared to normal cognition or MCI subjects. The total NPI score is significantly higher in the subjects of dementia (Figure 1). In addition, mood-related symptoms appear most frequently. Mood symptoms were observed in 124 participants (12.7%) with normal cognition, 131 participants (48.5%) with MCI, and 459 participants (70.7%) with dementia, indicating a significant increase in prevalence with cognitive decline (*p*-value < 0.001). Psychosis symptoms were present in 123 participants (12.6%) with normal cognition, 112 participants (41.5%) with MCI, and 359 participants (55.3%) with dementia, showing a similar trend of increasing frequency with worsening cognitive status (*p*-value < 0.001). Frontal symptoms were the least common, found in 29 participants (3.0%) with normal cognition, 45 participants (16.7%) with MCI, and 166 participants (25.6%) with dementia, again demonstrating an upward trend as cognitive impairment progresses (*p*-value < 0.001). Figure 2 indicates the distribution of the NPI-group score among three groups. 

### 2.3. Distribution of Cerebrospinal Fluid (CSF) Biomarkers

**Figure 3:**
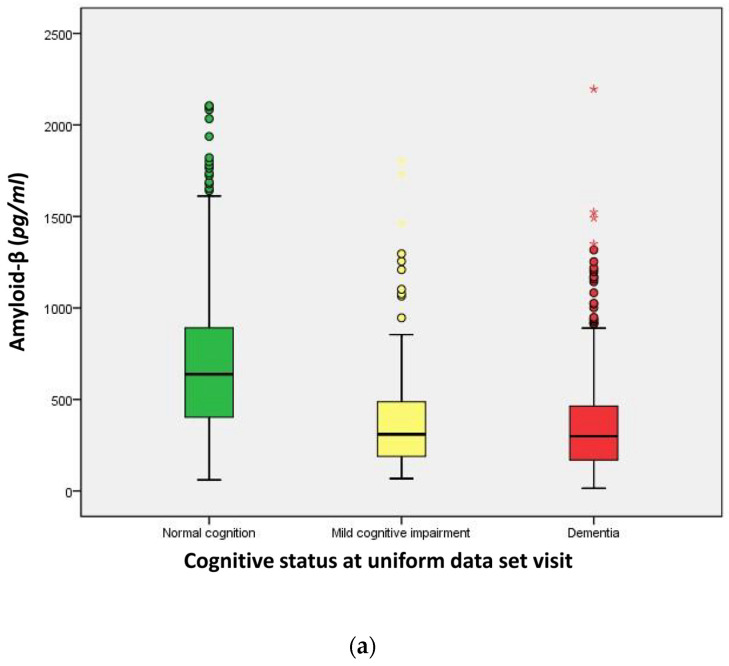

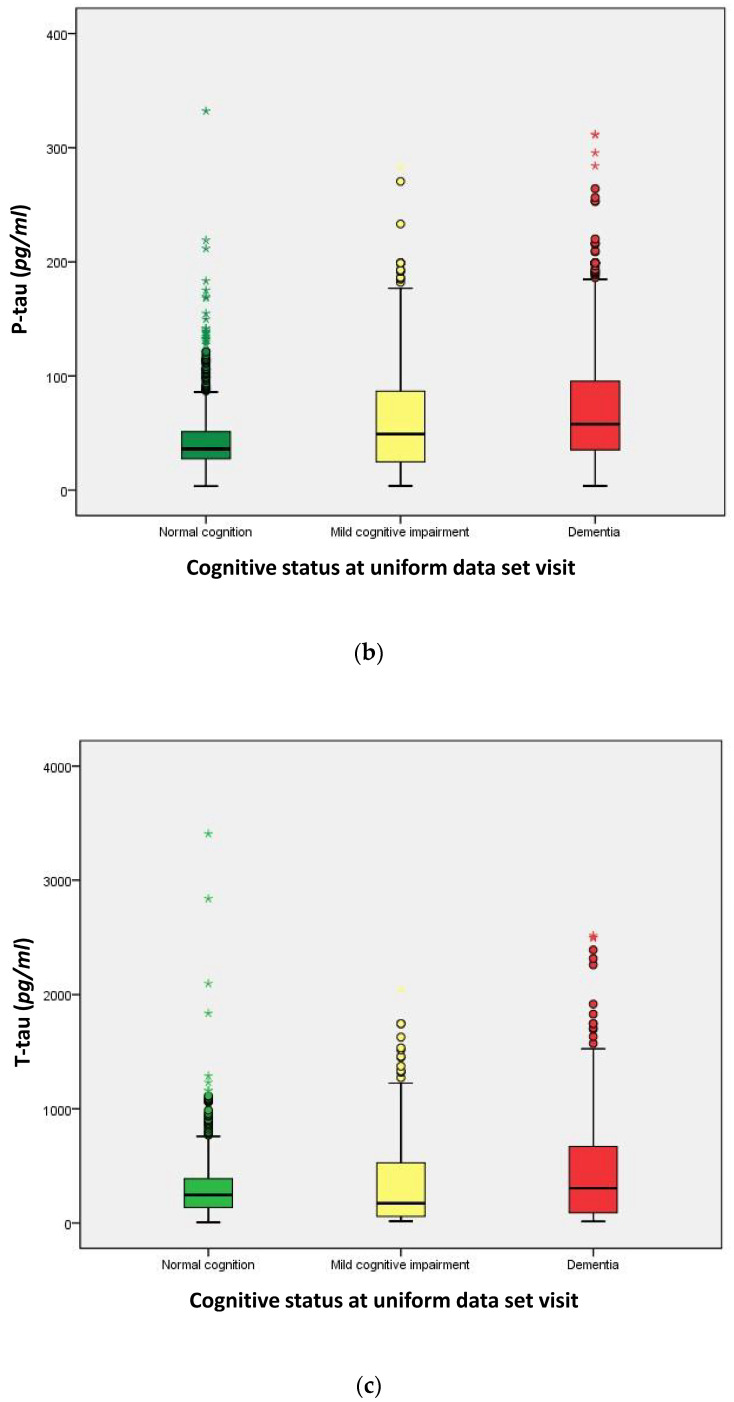

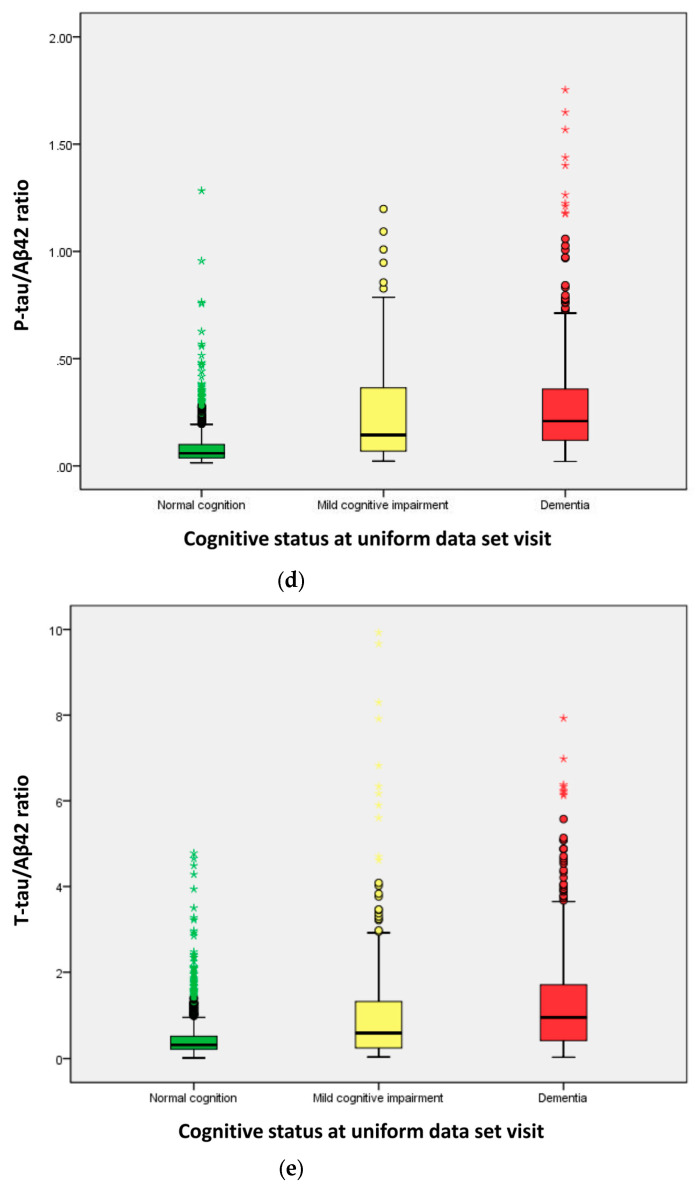
Distribution of CSF biomarkers. (**a**) Amyloid-β, (**b**) P-tau, (**c**) T-tau, (**d**) P-tau/Aβ, (**e**) T-tau/Aβ stratified by cognitive status. o (circle): Mild outliers, data points that are more extreme than than Q3 + 1.5 * IQR, but are not extreme outliers. * (asterisk): Extreme outliers, data points that are more extreme than Q3 + 3 * IQR.

The distributions of Amyloid-β, P-tau, and T-tau across the three distinct cognitive groups—normal cognition, MCI, and dementia—were markedly divergent (Kruskal–Wallis test, *p* < 0.001 for all). Specifically, the median Amyloid-β concentration was 656.0 pg/mL in individuals with normal cognition, compared to 300.6 pg/mL in those with MCI, and 298.8 pg/mL in dementia patients. Median P-tau levels were measured at 36.00 pg/mL for the normal cognition group, 49.12 pg/mL for the MCI group, and 58.29 pg/mL for the dementia group. Similarly, median T-tau concentrations were 241.0 pg/mL in the normal cognition group, 140.6 pg/mL in the MCI group, and 298.3 pg/mL in the dementia group. Notably, the T-tau/Aβ-42 ratio escalated from 0.058 in the normal cognition group to 0.144 in the MCI group, and further to 0.209 in the dementia group (*p* < 0.001). A parallel increase was observed in the P-tau/Aβ-42 ratio, starting at 0.305 in the normal cognition group, rising to 0.560 in the MCI group, and reaching 0.941 in the dementia group (*p* < 0.001) (Table 2, Figure 3). 

### 2.4. Prediction of Dementia

**Figure 4:**
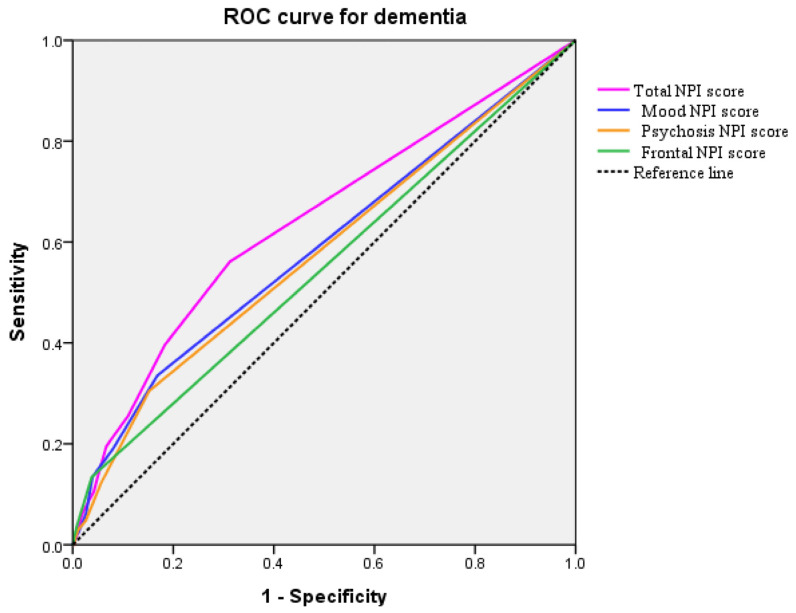
The ROC curve analysis of the Total NPI scores accuracy for Dementia prediction.

The ROC curves generated for the total NPI and three NPI-group scores were presented in Figure 4. Areas under the curve for the total NPI, mood, psychosis, and frontal symptom score were 0.638 (95% confidence interval 0.589–0.687, *p* < 0.001), 0.587 (0.536–0.638, *p* < 0.001), 0.577 (0.526–0.627, *p* = 0.002), and 0.548 (0.498–0.599, *p* = 0.005), respectively, indicate a moderate accuracy to predict dementia. 

**Figure 5:**
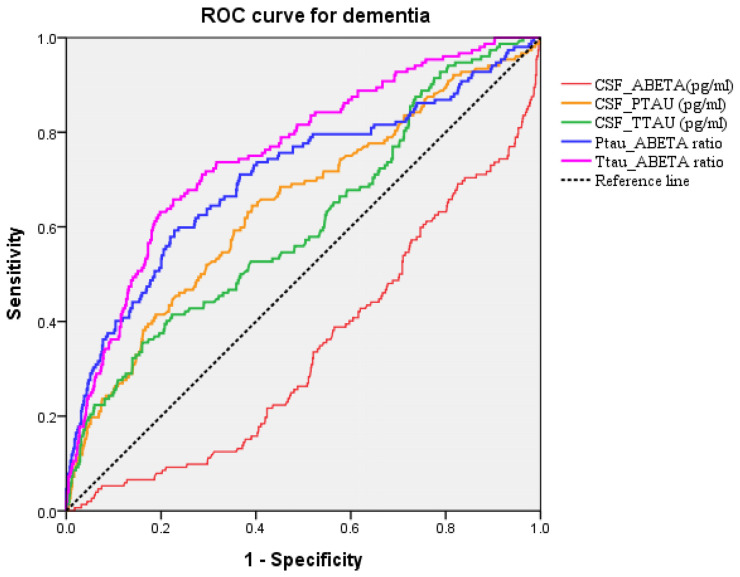
The ROC curve analysis of the CSF biomarkers accuracy for Dementia prediction.

The ROC curves generated for the CSF biomarkers are presented in Figure 5. AUC for the Amyloid-β, P-tau, T-tau, P-tau/Aβ42, and T-tau/Aβ42 were 0.334 (0.296–0.390, *p* < 0.001), 0.644 (0.594–0.695, *p* < 0.001), 0.607 (0.556–0.657, *p* < 0.001), 0.706 (0.655–0.756, *p* < 0.001) and 0.756 (0.714–0.799, *p* < 0.001), respectively, indicate a moderate accuracy to predict dementia. 

## 3. Methods

### 3.1. Data Source

Data from the NACC Version 3 of the Uniform Data Set (UDS) were used for this analysis. The NACC program was developed in 1999 and maintained by the National Institute on Aging to facilitate collaborative involvement among more than 42 Alzheimer’s Disease Research Centers (ADRCs) in the United States. The UDS forms were used to collect standardized clinical data from subjects who are evaluated on an approximately annual basis. Researchers in the ADRCs started submitting UDS data in September 2005. In 2015, the ADRCs implemented Version 3 of the UDS. NACC is one of the largest, oldest, and most powerful Alzheimer’s datasets. Further detailed information can be found elsewhere [40] or on the website of NACC accessed on 31 August 2020. https://naccdata.org/nacc-collaborations/about-nacc.

### 3.2. Ethics Statement

The NACC data were de-identified. The researcher using the NACC database was approved by the University of Washington Institutional Review Board [41]. Informed consent was obtained from all participants at the individual ADRC.

### 3.3. Study Subjects

Participants enrolled in the NACC program and with cognitive status ranging from normal cognition to MC), or dementia (which was determined based on an experienced clinician’s diagnosis and/or neuropsychological examination) were included in the present study [42]. Subjects were excluded if they never administered a Neuropsychiatry Inventory (NPI) evaluation, or if the results of the NPI evaluation were unknown or without CSF information. The duration between the date of the CSF test and the date of the NPI assessment is more than 3 years was also excluded.

Diagnoses within this framework are derived from comprehensive data collection by clinicians and clinic personnel trained in evaluating neurological and psychiatric conditions. This training includes but is not limited to the Clinical Dementia Rating (CDR) provided by the Washington University ADC. Diagnoses of dementia or MCI are made based on clinical symptoms observed and documented during the UDS annual visits, which cover a wide range of assessments from subject demographics to detailed neurological examinations. The ADRCs employ either a consensus team or a single physician, who has conducted the examination, to make these diagnoses, ensuring a consistent and accurate diagnostic process. For the diagnosis of dementia syndromes, our clinicians rely primarily on their clinical experience and observations of clinical signs, as outlined in the NACC guidebooks and staging instruments. These resources provide detailed guidelines for diagnosing dementia, emphasizing the importance of clinical symptoms over biomarkers or imaging results. Similarly, the diagnosis of MCI follows a structured process, with clinicians using medical records that include a neurologic examination, an imaging study, and cognitive testing (e.g., MMSE, Blessed, or more formal tests) to make informed decisions. In cases of uncertainty, additional information from co-participants and further review of clinical observations are utilized to confirm the diagnosis.

**Figure 6:**
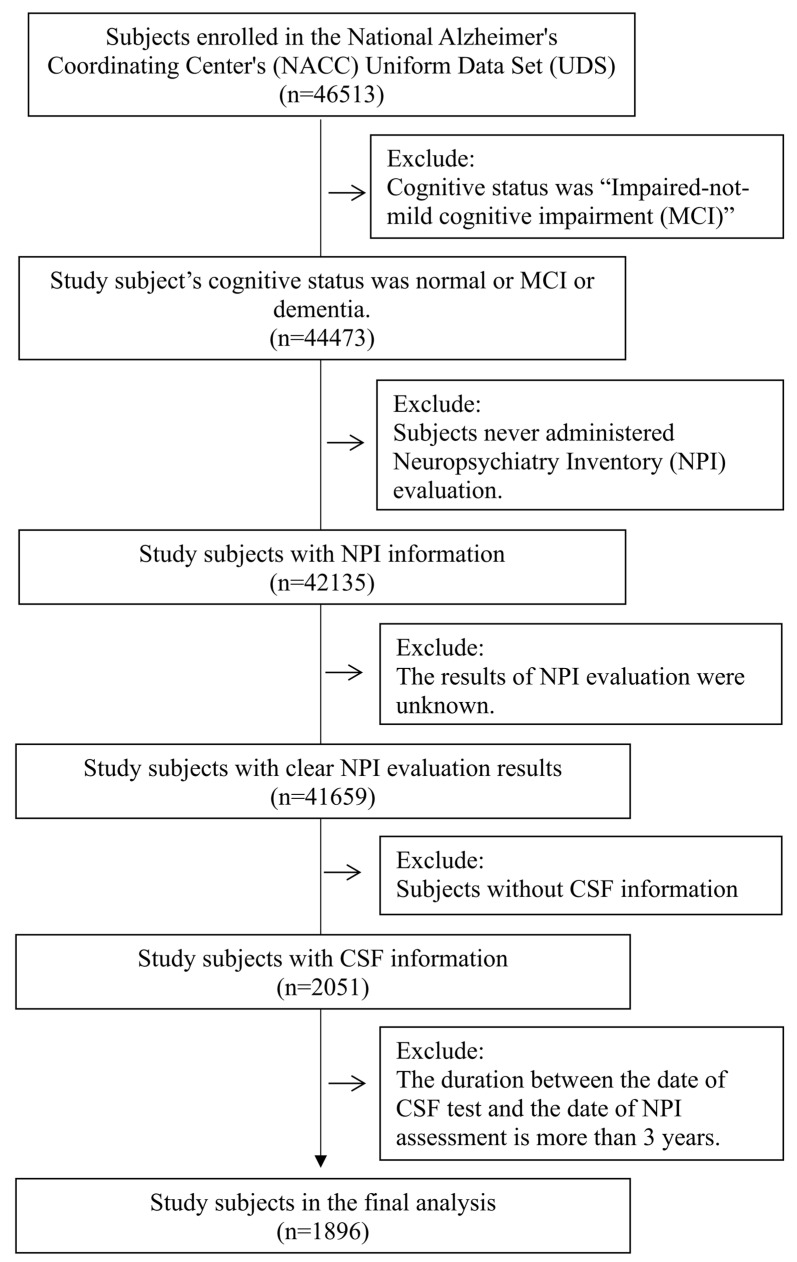
Flow chart of subject selection.

Participants were assigned as “cognitively normal” if they lacked significant cognitive or functional impairment; or “MCI” if they had objective or subjective evidence of cognitive impairment but no significant functional impairment to meet the criteria for dementia; or “dementia” if they had significant cognitive and functional impairment. Figure 6 indicates the selection process.

### 3.4. Study Variables

#### 3.4.1. Neuropsychiatry Inventory (NPI)

The Neuropsychiatric Inventory (NPI) is an essential tool in the field of neuropsychiatry for assessing the breadth and depth of behavioral disturbances associated with dementia. Its comprehensive approach evaluates a range of symptoms across 12 distinct domains, making it highly valuable for clinicians and researchers alike. The domains include delusions, hallucinations, agitation or aggression, depression or dysphoria, anxiety, elation or euphoria, apathy or indifference, disinhibition, irritability or lability, motor disturbance, nighttime behaviors, and appetite and eating problems. This broad spectrum allows for a nuanced understanding of the patient’s neuropsychiatric profile, facilitating targeted interventions [43]. 

The NPI-Questionnaire (NPI-Q) serves as a streamlined version of the NPI [44], designed for efficient clinical use while maintaining the integrity of the original assessment’s objectives. It simplifies the process of gathering information on the prevalence and severity of the 12 neuropsychiatric symptoms, either from the subjects themselves or their caregivers. The severity of these symptoms is rated on a three-point scale (1 = mild, 2 = moderate, 3 = severe), with the total NPI score ranging from 0 to 36. This quantification allows for the objective measurement of symptom severity and can guide treatment planning and monitoring over time.

Further, the NPI-Q categorizes symptoms into three groups to enhance the clinical utility and interpretation of the data: mood, psychosis symptoms, and frontal symptoms. The mood category includes anxiety, apathy, and dysphoria, with a score range of 0 to 9. The psychosis symptoms group encompasses irritability/lability, delusions, hallucinations, and agitation/aggression, offering a score range of 0 to 12. The frontal symptoms category, which includes euphoria and disinhibition, has a score range of 0 to 6. This categorization helps in identifying the predominant symptomatology, facilitating a more focused therapeutic approach.

The application of the NPI and NPI-Q in clinical settings and research, such as within the National Alzheimer’s Coordinating Center Uniform Data Set (NACC UDS), underscores their importance in understanding the complex interplay of neuropsychiatric symptoms in dementia. By providing a structured and reliable method for evaluating these symptoms, the NPI and NPI-Q contribute significantly to the personalized care of individuals with dementia, enabling clinicians to address not only the cognitive aspects of the disease but also the challenging behavioral and psychological symptoms that affect the quality of life for patients and their families.

#### 3.4.2. Cerebrospinal Fluid (CSF) Biomarker

The CSF biomarkers for Amyloid-β42, P-tau, and T-tau were analyzed using the enzyme-linked immunosorbent assay (ELISA) or Luminex multiplex xMAP assay protocols.

1. Amyloid-β42 (Aβ42): Aβ42 plays a pivotal role in the pathogenesis of AD, and its levels in the CSF have been closely linked with the disease’s progression and diagnosis. The aggregation of Aβ42 into amyloid plaques in the brain is a hallmark of AD. These plaques disrupt cell-to-cell communication and activate immune responses, which can lead to inflammation and the destruction of neurons. The measurement of Aβ42 levels in the CSF provides an indirect marker of plaque burden within the brain. Importantly, reductions in CSF Aβ42 levels can occur years before the onset of clinical symptoms, making it a potential biomarker for early detection and intervention in AD. Additionally, Aβ42 levels are being explored in the context of clinical trials for amyloid-targeting therapies, serving as a biological endpoint for disease-modifying treatments.
2. Phosphorylated tau (P-tau): P-tau is a specific marker for AD pathology. The hyperphosphorylation of tau protein leads to its aggregation into neurofibrillary tangles, another pathological hallmark of AD. These tangles accumulate inside neurons, disrupting their function and eventually leading to cell death. The levels of P-tau in the CSF are correlated with the presence and progression of tau pathology in the brain. Elevated P-tau levels are considered a sign of ongoing neurodegenerative processes and have been associated with cognitive decline and the severity of AD. P-tau levels in the CSF are also being studied as a biomarker for tracking disease progression and response to tau-targeted therapies.
3. Total tau (T-tau): T-tau in the CSF is a marker of neuronal damage and neurodegeneration. Unlike Aβ42 and P-tau, which are more specific to AD pathology, elevated T-tau levels can be seen in various conditions that cause neuronal damage, such as traumatic brain injury, stroke, and other neurodegenerative diseases. This makes T-tau a less specific biomarker for AD but valuable in the broader context of neurological damage assessment. In AD, the combination of high T-tau and P-tau levels with low Aβ42 levels in the CSF can significantly enhance diagnostic accuracy, differentiating AD from other forms of dementia and neurodegenerative disorders.

Together, the measurement of Aβ42, P-tau, and T-tau levels in the CSF provides a powerful toolset for the early diagnosis, differentiation, and monitoring of AD and other neurodegenerative conditions. These biomarkers not only aid in understanding the underlying mechanisms of these diseases but also play a crucial role in the development and evaluation of targeted therapies, offering hope for improved outcomes for affected individuals. The specific patterns of these biomarkers—decreased Aβ42, with increased P-tau and T-tau—support the diagnosis of AD, offering insights into the disease’s pathophysiology and progression.

### 3.5. Covariates 

Demographic variables such as age at visit (≦65 y, 66–75 y, ≧76 y), sex, race/ethnicity, education level, marital status, living situation, and first-degree family member with cognitive impairment were obtained from the NACC database. Race was grouped as White, Black or African American, and others. Marital status was categorized as married/living as a married/domestic partner, widowed/divorced/separated, and never married. Educational status was grouped as high school, bachelor’s, and master’s or PhD. The living situation was categorized as living alone or living with others. 

Lifestyle/Physical function variables included smoking, alcohol abuse, other substance abuse, level of independence, body mass index (BMI), and vision and hearing function. Participants’ smoking status was divided into two groups: non-smokers who reported having never smoked at least 100 cigarettes in their lifetime, and smokers who had smoked over 100 cigarettes during their lifetime. A subject-reported clinical impairment that has significant negative effects on work, driving, legal, or social aspects over a 12-month period was considered the definition of alcohol abuse in the present study. The level of independence was classified into two categories: able to live independently, and dependent (requiring some assistance or being completely dependent). According to the WHO criteria, body mass index (BMI) was classified into three groups: underweight (<18.5 kg/m^2^), normal or overweight (18.5~29.9 kg/m^2^), and obese (≥30.0 kg/m^2^). The subject’s vision was considered functionally normal based on self-report, whether corrective lenses were worn or not. Subject’s hearing was considered functionally normal also based on self-report, whether hearing aid(s) were worn or not.

Comorbidities, such as cardiovascular disease (CVD, including heart attack, atrial fibrillation, congestive heart failure, angina, myocardial infarction, other cardiovascular disease, or administered angioplasty/endarterectomy/stent, percutaneous coronary intervention, cardiac bypass procedure, pacemaker or defibrillator, or heart valve replacement or repair), stroke/transient ischemic attack (TIA), Parkinson’s disease (PD) or other parkinsonian disorder, traumatic brain injury (TBI, including brain trauma, chronic traumatic encephalopathy), diabetes, hypertension, hypercholesterolemia, vitamin B12 deficiency, thyroid disease, arthritis, sleep disorders (including apnea, rapid eye movement sleep behavior disorder, hyposomnia or insomnia, other sleep disorder), psychological diseases (including post-traumatic stress disorder, bipolar disorder, schizophrenia, obsessive–compulsive disorder, developmental neuropsychiatric disorders (such as autism spectrum disorder, attention-deficit hyperactivity disorder, or dyslexia), and other psychiatric disorder), and cancers were incorporated into present study. This comorbidity information was collected using mainly subject and co-participant reports. 

Medication variables mainly collect the current usage (two weeks of UDS visit) of the following medications: nonsteroidal anti-inflammatory medication, anticoagulant or antiplatelet agent, antipsychotic/anxiolytic, sedative, or hypnotic agent, antiparkinson agent, hormone therapy, antihypertensive or blood pressure, lipid-lowering medication, diabetes medication, and antidepressant. Details of medications included in each category were described in Version 3 of the UDS.

### 3.6. Statistical Analyses

Data of basic characteristics were expressed as counts (%) for categorical variables and median for continuous variables. A chi-square test was conducted to determine the differences in categorical variables and differences in continuous variables were examined using the Kruskal–Wallis test due to the data not being normally distributed. 

To depict the distribution of the outcomes of interest, box plots were used to analyze the total NPI score, as well as three groups of NPI scores and CSF biomarkers. In order to assess the precision of the total NPI scores in predicting dementia, an analysis of the receiver operating characteristic (ROC) curve and area under the curve (AUC) was conducted. The ROC curve is a graphical representation of the sensitivity (true positive rate) versus 1-specificity (false positive rate) of total NPI score across a range of decision thresholds. The AUC is a summary measure of the overall prediction accuracy of the score. 

All statistical assessments were two-sided and evaluated at the 0.05 level of significance. Statistical analyses were performed using the statistical software package SPSS complex sample module version 22.0 (IBM Corp, Armonk, NY, USA).

## 4. Discussion

Our study utilized the comprehensive dataset from the NACC’s UDS Version 3, engaging a broad cohort of 1896 participants. The diversity and size of our sample enhance the generalizability of our findings, laying a foundation for longitudinal investigations that could elucidate the causal relationships among neuropsychiatric symptoms, CSF biomarkers, and cognitive transitions. The cross-sectional nature of our analysis initiates an exploration of these complex interactions, aiming to refine clinical assessments and improve patient outcomes. We sought to bridge a significant gap in existing research by correlating neuropsychiatric symptoms, as quantified by the NPI, with key CSF biomarker levels across varying cognitive states. Our findings reveal notable differences in both NPI scores and CSF biomarker levels among individuals with normal cognition, MCI, and AD, supporting the hypothesis that integrating these assessments can enhance early diagnostic processes. This contribution is not merely a recapitulation of known facts but extends our understanding of the diagnostic value of CSF biomarker ratios, potentially offering new directions for AD’s diagnostics.

The incorporation of CSF biomarkers with NPI scores aims to establish a comprehensive diagnostic framework. This integration seeks to combine the biological markers of neuropathological change with the psychological and behavioral dimensions of cognitive impairment, thereby aiming to heighten the early detection accuracy for MCI and AD [45]. Our analysis indicates that such a multifaceted approach could allow for earlier and more precise identification of individuals at risk, beyond what might be achieved through singular assessment methodologies.

Significant variations in median Amyloid-β, P-tau, and T-tau levels across cognitive groups underscore the potential of these biomarkers in reflecting the disease’s progression. These observations align with prior research indicating a link between neuropsychiatric symptom escalation and cognitive decline transitions [46,47,48,49], as well as studies affirming the relationship between CSF biomarker alterations and cognitive deterioration [50,51]. However, it is crucial to interpret these findings within the context of association rather than causality, as our study primarily underscores the significant correlations without asserting direct causal links. This distinction is vital for understanding the broader implications of our findings within the scope of cognitive impairment research.

Extending beyond mere associations, our study delves into the evolving patterns of CSF biomarkers and their interrelations with cognitive states, offering invaluable insights for both diagnosis and prognosis in cognitive impairment and dementia contexts. This depth of analysis surpasses the scope of Krell-Roesch’s investigation [36], providing a comprehensive overview of the cognitive spectrum that facilitates a nuanced appreciation of cognitive decline’s progression and implications. The observed variations in NPI and CSF biomarkers suggest the feasibility of their combined use in early MCI and AD diagnostics, which could enhance diagnostic precision and facilitate timely intervention strategies [45].

## 5. Future Directions

This study lays a foundational understanding of the correlations between neuropsychiatric symptoms, CSF biomarkers, and cognitive states across a spectrum from normal cognition to dementia. However, it also opens several avenues for future research. First, longitudinal studies are crucial to establishing causal relationships between these variables, providing deeper insights into the progression from MCI to AD and potentially identifying early intervention points. Furthermore, expanding the biomarker profile to include other relevant markers, such as Amyloid-β40, would enrich our understanding of the neuropathological underpinnings of cognitive decline and dementia.

Investigations into the impact of various interventions, including pharmacological, lifestyle, and cognitive rehabilitation measures, on the progression of MCI to AD could offer valuable information on mitigating the disease’s impact. Additionally, exploring the genetic and environmental factors contributing to the variability in neuropsychiatric symptoms and biomarker levels among individuals could help tailor more personalized and effective treatment approaches.

## 6. Limitations

Our study has several limitations that need to be acknowledged. First, the cross-sectional design of the study limits our ability to establish causality between the observed variations in NPI and CSF biomarkers and the progression from normal cognition to MCI and AD. Longitudinal studies are needed to confirm the causal relationships and to examine the potential predictive value of these measures over time. Second, our study population consisted of individuals from the NACC, which may limit the generalizability of our findings to other populations. NACC subjects are not a statistically based sample of the U.S. population—with or without dementia. Rather, they are best regarded as a referral-based or volunteer case series. Third, the NPI assesses a limited range of neuropsychiatric symptoms, and other unmeasured symptoms or factors may also play a crucial role in the progression from normal cognition to MCI and AD. Fourth, our study did not incorporate detailed information on potential comorbidities, such as Lewy Body disease and cerebrovascular burden, which could have nuanced our understanding of the observed neuropsychiatric symptoms. Fifth, the effects of medication, sleep disorders (including obstructive sleep apnea, which has been linked to amyloid deposition and cognitive decline), and conditions like epilepsy were not examined due to data limitations. Additionally, our study did not capture detailed information regarding the pharmacological treatments administered to individuals across different cognitive statuses. This omission limits our ability to assess how specific drugs or therapeutic strategies might influence NPI in the context of AD and other cognitive impairments. Notably, certain medications known for their symptomatic effects could potentially offer benefits in alleviating these symptoms, particularly in the early stages of cognitive decline. The absence of these data constrains our insight into the potential therapeutic impacts on NPI outcomes, highlighting a critical area for future research to explore in order to enhance treatment efficacy and patient quality of life.

## 7. Conclusions

Our study revealed significant variations in the NPI and CSF biomarkers among individuals with normal cognition, MCI, and AD. These findings suggest that combining the assessment of neuropsychiatric symptoms and key CSF biomarkers may improve the early diagnosis of MCI and AD and enable timely interventions. Further studies are needed to confirm these findings and to explore the potential implications for clinical practice and the development of innovative intervention strategies.

## Figures and Tables

**Table 1:** Characteristics of participants.

Variables	Normal Cognition (n = 977)	MCI (n = 270)	Dementia (n = 649)	*p* Value
**Demography**				
Age (year, n, %)				0.045
<65 y	197 (20.2)	54 (20.0)	163 (25.1)	
≥65 y	780 (79.8)	216 (80.0)	486 (74.9)	
Sex (n, %)				<0.001
Male	418 (42.8)	156 (57.8)	342 (52.7)	
Female	559 (57.2)	114 (42.2)	307 (47.3)	
Race/Ethnicity (n, %)				0.027
White	860 (88.0)	241 (89.3)	576 (88.8)	
Black/African American	71 (0.73)	8 (0.30)	35 (0.54)	
Others	46 (0.47)	21 (0.78)	38 (0.59)	
Education (n, %)				<0.001
High school or less	134 (13.7)	62 (23.0)	202 (31.3)	
Bachelor degree	412 (42.3)	103 (38.3)	265 (41.1)	
Master’s or doctoral degree	429 (44.0)	104 (38.7)	178 (27.6)	
Marital status (n, %)				<0.001
Married	679 (69.6)	200 (74.1)	516 (79.5)	
Widowed/divorced/separated	241 (24.7)	63 (23.3)	112 (17.3)	
Never married	55 (0.56)	7 (0.26)	21 (0.32)	
Living situation (n, %)				<0.001
Living alone	242 (24.8)	51 (18.9)	85 (13.1)	
Living with others	735 (75.2)	219 (81.1)	564 (86.9)	
Family history (with dementia) (n, %)				<0.001
No	335 (34.3)	89 (33.0)	213 (32.8)	
Yes	612 (62.6)	155 (57.4)	382 (58.9)	
Unknown	30 (0.31)	26 (0.96)	54 (0.83)	
**Lifestyles/Physical status**				
Smoking				0.912
Yes	451 (46.7)	128 (47.9)	306 (47.5)	
Alcohol abuse				0.407
Yes	43 (0.44)	13 (0.48)	38 (0.59)	
Other substances abuse				0.412
Yes	9 (0.09)	2 (0.07)	10 (0.15)	
Level of independence				<0.001
Independent	969 (99.2)	185 (68.5)	209 (32.2)	
Dependent	8 (0.08)	85 (31.5)	440 (67.8)	
Body mass index (kg/m^2^, n, %)				0.820
<18.5	13 (0.13)	6 (0.22)	8 (0.12)	
18.5~29.9	722 (73.9)	198 (73.3)	484 (74.6)	
≧30	242 (24.8)	66 (24.4)	157 (24.2)	
Vision				<0.001
Functionally normal	939 (96.1)	247 (91.5)	586 (90.3)	
Hearing				0.461
Functionally normal	901 (92.2)	244 (90.4)	589 (90.8)	
**Comorbidities (Yes, n, %)**				
Cardiovascular disease	259 (26.5)	74 (27.4)	172 (26.5)	0.953
Stroke/Transient ischemic attack (TIA)	40 (0.41)	19 (0.70)	44 (0.68)	0.029
Parkinson’s disease (PD)	17 (0.17)	15 (0.56)	29 (0.45)	0.001
Seizures/Other neurological condition	23 (0.24)	8 (0.30)	26 (0.40)	0.161
Traumatic brain injury (TBI)	91 (0.93)	35 (13.0)	58 (0.89)	0.144
DM	99 (10.1)	49 (18.1)	69 (10.6)	0.001
Hypertension	443 (45.3)	146 (54.1)	311 (47.9)	0.038
Hypercholesterolemia	543 (55.6)	153 (56.7)	348 (53.6)	0.628
B12 deficiency	65 (0.67)	16 (0.59)	42 (0.65)	0.912
Thyroid disease	188 (19.2)	52 (19.3)	98 (15.1)	0.082
Arthritis	461 (47.2)	73 (27.0)	113 (17.4)	<0.001
Sleep disorders	268 (27.4)	66 (24.4)	100 (15.4)	<0.001
Psychological disease	35 (0.36)	20 (0.74)	38 (0.59)	0.014
Cancer	102 (10.4)	26 (0.96)	30 (0.46)	<0.001
**Medication usage**				
NSAIDs	410 (42.0)	113 (41.9)	213 (32.8)	0.001
Anticoagulant or antiplatelet agent	322 (33.0)	92 (34.1)	190 (29.3)	0.207
Antipsychotic agent	122 (12.5)	48 (17.8)	110 (16.9)	0.015
Antiparkinson agent	43 (0.44)	19 (0.70)	23 (0.35)	0.065
Hormone therapy	50 (0.51)	6 (0.22)	11 (0.17)	0.001
Antihypertensive or blood pressure	434 (44.4)	149 (55.2)	328 (50.5)	0.002
Lipid-lowering medication	384 (39.3)	125 (46.3)	294 (45.3)	0.021
Diabetes medication	72 (0.74)	36 (13.3)	49 (0.76)	0.005
Antidepressant	218 (22.3)	93 (34.4)	261 (40.2)	<0.001

MCI: Mild cognitive impairment. NSAIDs: Nonsteroidal anti-inflammatory medication.

**Table 2:** Distribution of neuropsychological function (Neuropsychiatric Inventory Questionnaire) and CSF biomarkers.

Variables	Normal Cognition (n = 977)	MCI (n = 270)	Dementia (n = 649)	*p* Value
**NPI (Yes, n, %)**				
Delusions	5 (00.5)	14 (05.2)	97 (14.9)	<0.001
Hallucinations	2 (00.2)	7 (02.6)	43 (06.6)	<0.001
Agitation	49 (05.0)	56 (20.7)	227 (35.0)	<0.001
Depression	93 (09.5)	86 (31.9)	284 (43.8)	<0.001
Anxiety	44 (04.5)	70 (25.9)	247 (38.1)	<0.001
Elation	8 (00.8)	10 (03.7)	31 (04.8)	<0.001
Apathy	26 (02.7)	62 (23.0)	294 (45.3)	<0.001
Disinhibition	24 (02.5)	41 (15.2)	151 (23.3)	<0.001
Irritability	96 (09.8)	81 (30.0)	257 (39.6)	<0.001
Aberrant motor behavior	6 (00.6)	17 (06.3)	117 (18.0)	<0.001
Night-time behavior	80 (08.2)	58 (21.5)	173 (26.7)	<0.001
Appetite	48 (04.9)	36 (13.3)	161 (24.8)	<0.001
**NPI-group (Yes, n, %)**				
Mood symptoms	124 (12.7)	131 (48.5)	459 (70.7)	<0.001
Psychosis symptoms	123 (12.6)	112 (41.5)	359 (55.3)	<0.001
Frontal symptoms	29 (03.0)	45 (16.7)	166 (25.6)	<0.001
**Total NPI score (median)**	0.00	2.00	4.00	<0.001 ^a^
**NPI-group score (median)**				
Mood symptoms score	0.00	0.00	2.00	<0.001 ^a^
Psychosis symptoms score	0.00	0.00	1.00	<0.001 ^a^
Frontal symptoms score	0.00	0.00	0.00	<0.001 ^a^
**CSF biomarkers (median)**				
Amyloid-β (Aβ_42_, (pg/mL))	656.0	300.6	298.8	<0.001 ^a^
P-tau181P (pg/mL)	36.00	49.12	58.29	<0.001 ^a^
T-tau (pg/mL)	241.0	140.6	298.3	<0.001 ^a^
P-tau/Aβ_42_ ratio	0.058	0.144	0.209	<0.001 ^a^
T-tau/Aβ_42_ ratio	0.305	0.560	0.941	<0.001 ^a^

a. Kruskal-Wallis test.

## Data Availability

The data supporting the findings of this study are not publicly available.
